# Supplementary material for: Integrative metagenomics and metabolomics reveal age-associated gut microbiota and metabolite alterations in a hamster model of COVID-19
Source: Gut Microbes. 2025 Apr 2;17(1):2486511. doi: 10.1080/19490976.2025.2486511 (PMC11970752; doi:10.1080/19490976.2025.2486511)
Supplement: Supplemental Material [file KGMI_A_2486511_SM9358.docx]

**Supplementary Figure 1. Alpha diversity and taxonomy of gut microbiota and plasma metabolite composition in young and aged hamsters (steady state). a,** Alpha diversity analysis of gut microbiota communities (genus level). Boxplots representing the distribution of the Shannon diversity index for young (light blue) and aged (dark blue) hamsters. **b**, Bar plots showing the distribution of bacterial genera within each sample group. **c,** Heatmap displaying the top 50 metabolites selected based on raw P-value. Clustering was performed using the average method with Euclidean distances as the metric. Significant differences were determined using the T-test (**P* < 0.05). Each colored cell on the map corresponds to a normalized metabolite concentration, with metabolites name in rows and samples in columns. Young=light blue; Aged=dark blue.

**Supplementary Figure 2. Body weight loss and recovery, expression of fibrotic markers (D22) and lung transcriptomic signatures (D7) of SARS-CoV-2-infected young and aged hamsters. a**, Schematic representation of the experimental design. **b**, *Left* panel: Percentage of body weight loss in SARS-CoV-2-infected young and aged hamsters at D8. *Right* panel: Percentage of body weight gain from D8 onwards (6 young hamsters and 3 aged hamsters). **c** and **d**, Lungs from vehicle-treated (mock) and SARS-CoV-2-infeced young and aged were collected on D7. mRNA copy numbers (for ISGs and inflammatory genes in panel **c** and for genes related to barrier functions in panel **c**) were quantified by RT-PCR. The data are expressed as the mean fold change relative to average gene expression in mock-infected young animals (n=6). **e**, Venn diagrams showing the distribution of up-regulated (n=2430) and down-regulated (n=3648) genes at D7. Compared to non-infected age-matched hamsters, 1,252 genes were up-regulated and 3,325 genes were down-regulated in the young group, whereas 2,153 genes were up-regulated and 1,988 were down-regulated in the aged group (*P* < 0.05). **f**, Gene set enrichment analysis from Kyoto Encyclopedia of Genes and Genomes (KEEG) and Keywords Biological Process (KW_BP) (common to both young and aged hamsters) (n=4/group). Regarding common genes shared between young and aged hamsters (40% of upregulated and 45% of downregulated genes, gene ontology (GO) enrichment analysis revealed that upregulated genes were associated with “Immunity”, “Cell cycle/cell division”, and “Complement and coagulation cascades”, while downregulated genes were related to signaling pathways such as “MAPK”, “Chemokine receptor”, “VEGF”, and “Notch signaling”. Significant differences were determined using the Mann Whitney *U* test (**b**, left panel, **c** and **d**) (**P* < 0.05, ***P* < 0.01).

**Supplementary Figure 3. Diversity and functional profile of gut microbiota across different age groups and infection states. a,** Alpha diversity (genus) analysis of gut microbiota communities. Boxplots representing the distribution of the Shannon diversity index for non-infected and infected young and aged hamsters are depicted. **b**, Bar plots showing the distribution of bacterial genera within each sample group. **c**, MaAsLin2 multivariate differential analysis for gut microbiota functional pathways as a heatmap, showing infection and age effects. Positive values (red) indicated enrichment in D22 relative to D7 groups or in aged relative to young groups.

**Supplementary Figure 4. Permutation test and global metabolic network of enrichment pathways from plasma metabolome of infected young and aged hamsters. a,** Validation by permutation tests based on separation ANOVA-simultaneous component analysis (ASCA). *P* value based on permutation for age, infection state, and their interaction (*P* <0.05). **b**, Pathway analysis based on metabolite sets enrichment performed with the list of significant metabolites (10), identifying the most relevant metabolic pathways via pathway impact and adjusted *P*-value. Figures were drawn using Metaboanalyst software v 5.0. **c,** Global metabolic network of Small Molecular Pathway Database (SMDB) pathways.

**Supplementary Figure 5. Metabolite concentration shifts in young hamsters post-SARS-CoV-2 infection: insights from network analysis of D7 and D22 post-infection.** Plasma concentration of host-related (**a**) and microbiota-related (**b**) metabolites. Data are expressed as mean ± SEM (*n* = 3-5). Significant differences were assessed using the Kruskal-Wallis test, a non-parametric one-way ANOVA, with multiple comparisons (D7 *vs* D0; D22 *vs* D0). Statistical significance is indicated as follows: *P < 0.05; **P < 0.005 ; ***P<0.001. The bars represent the mean ± SEM.

**Supplementary Figure 6. Metabolite concentration shifts in aged hamsters post-SARS-CoV-2 infection: insights from network analysis of D7 and D22 post-infection.** Plasma concentration of host-related (**a**) and microbiota-related (**b**) metabolites. Data are expressed as mean ± SEM (*n* = 3-5). Significant differences were assessed using the Kruskal-Wallis test, a non-parametric one-way ANOVA, with multiple comparisons (D7 *vs* D0; D22 *vs* D0). Statistical significance is indicated as follows: *P < 0.05; **P < 0.005 ; ***P<0.001. The bars represent the mean ± SEM.

**Supplementary Figure 1**

**
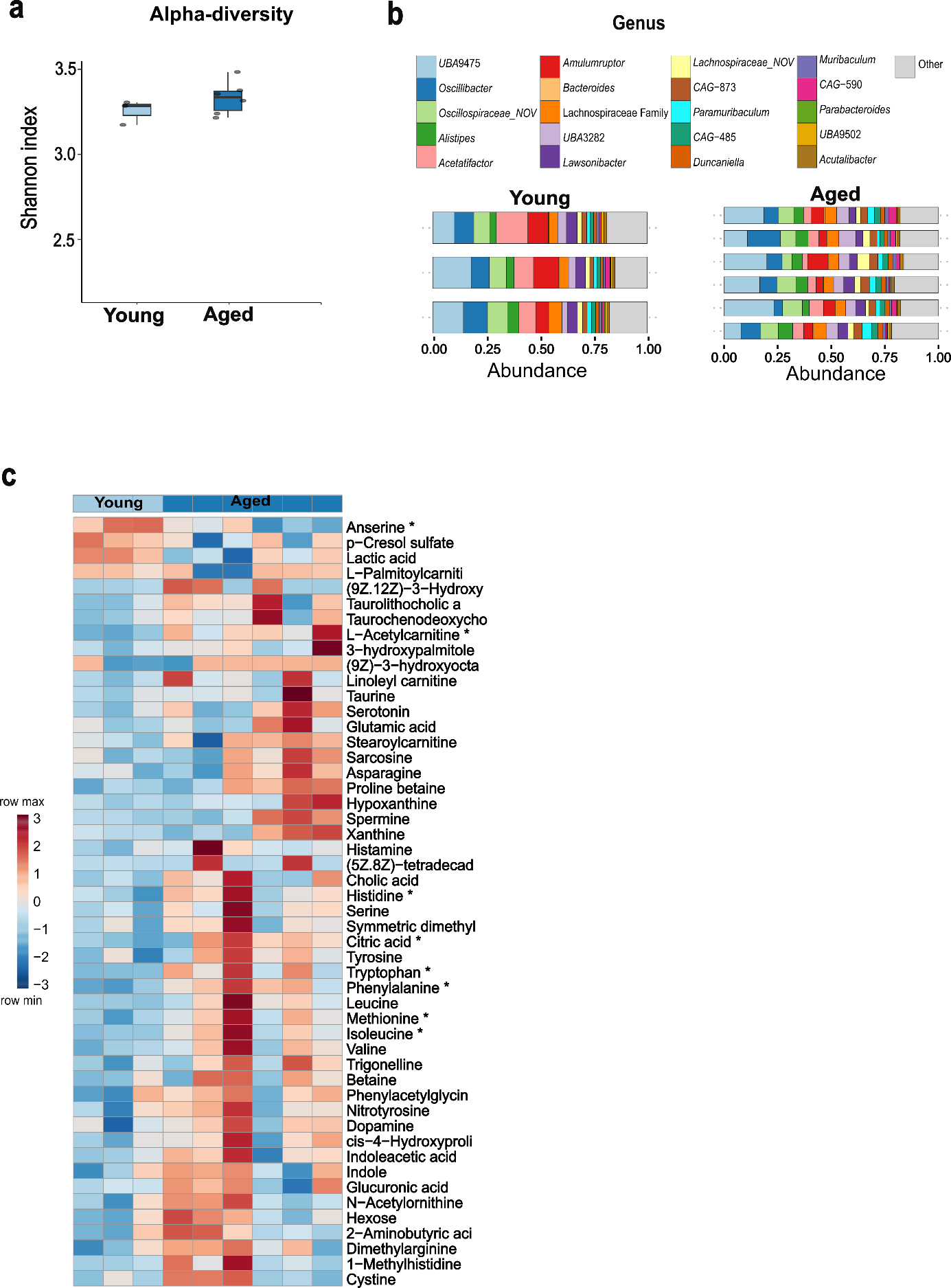
**

**Supplementary Figure 2**

**
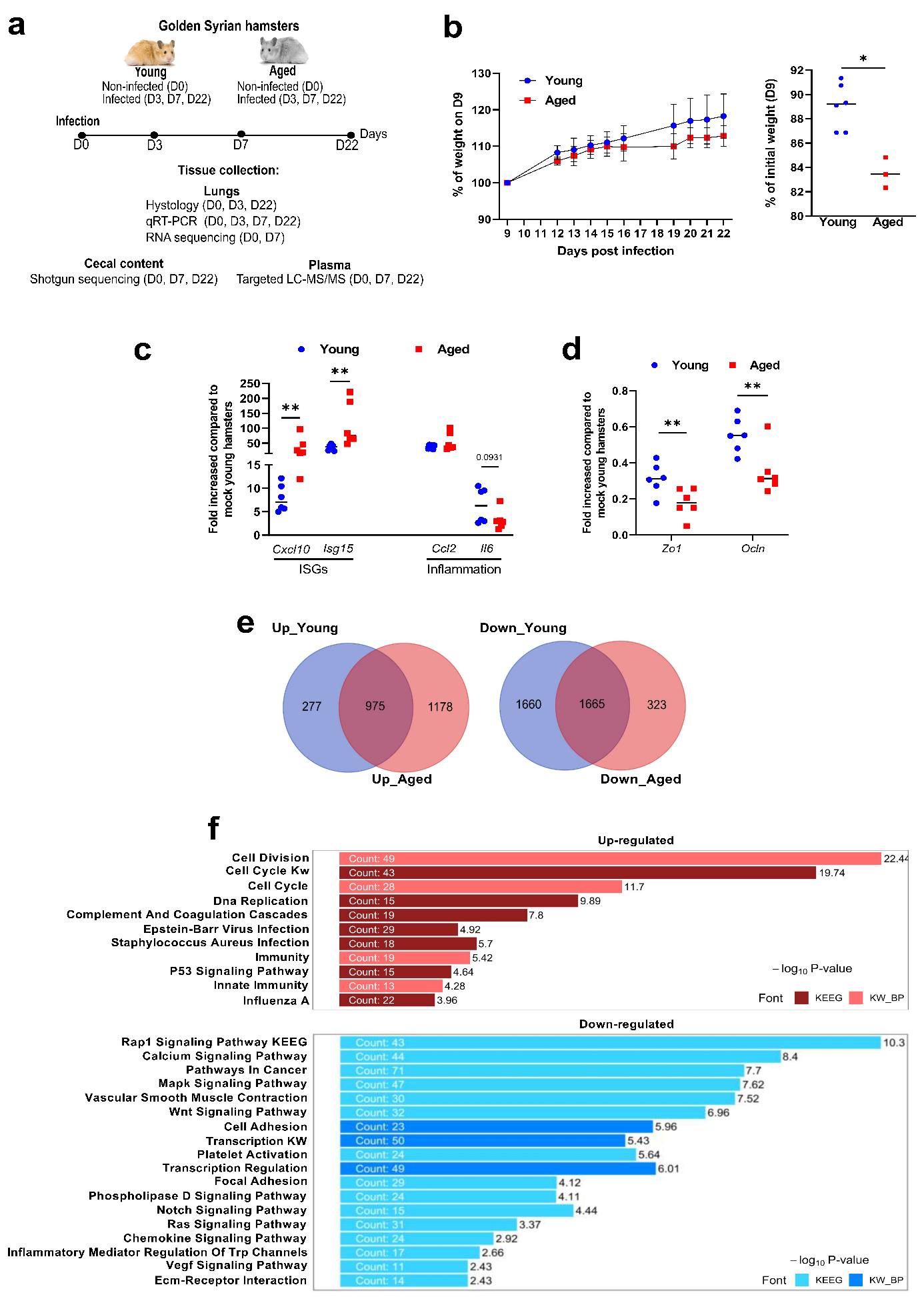
**

**Supplementary Figure 3**

**
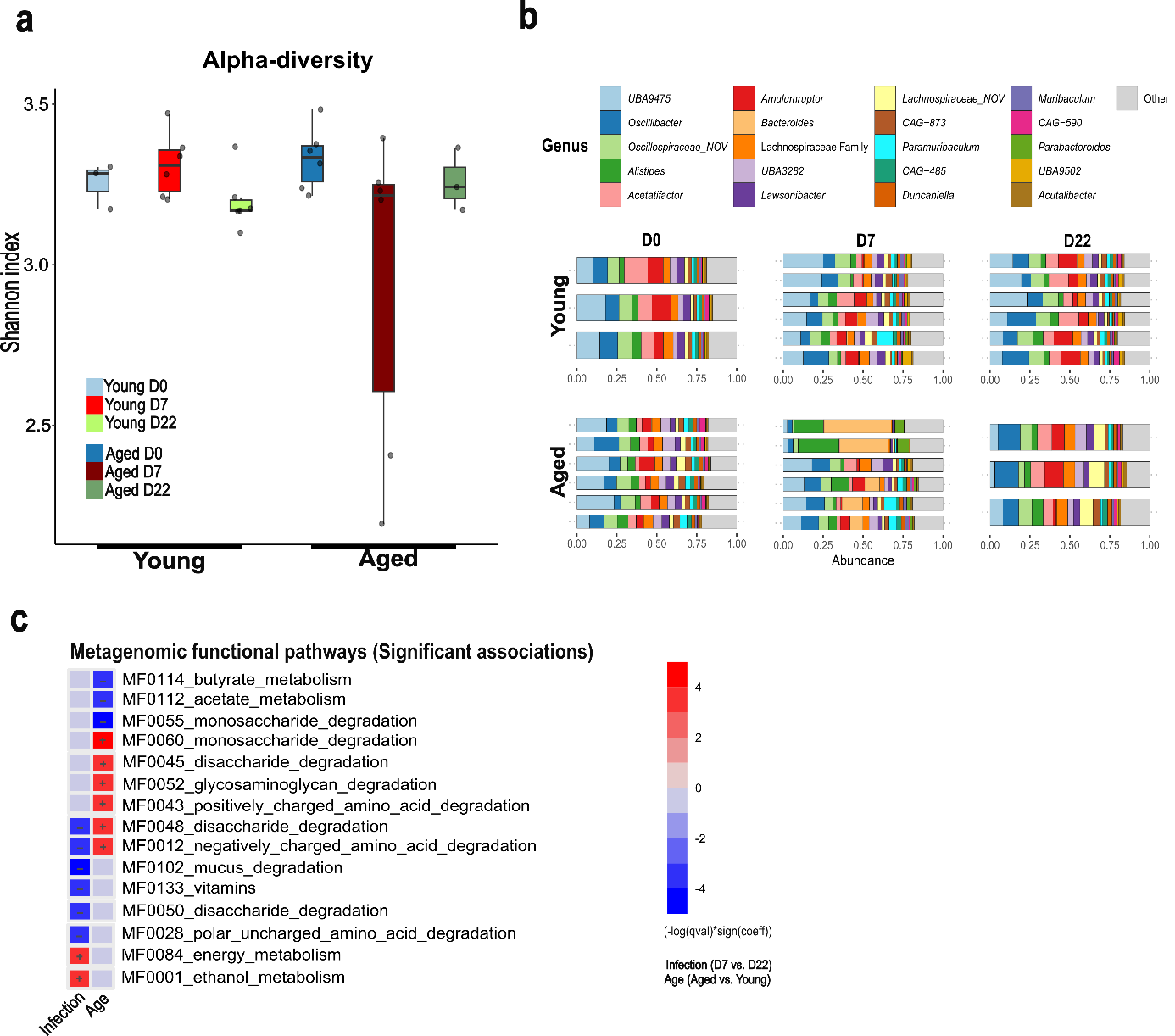
**

**Supplementary Figure 4**

**
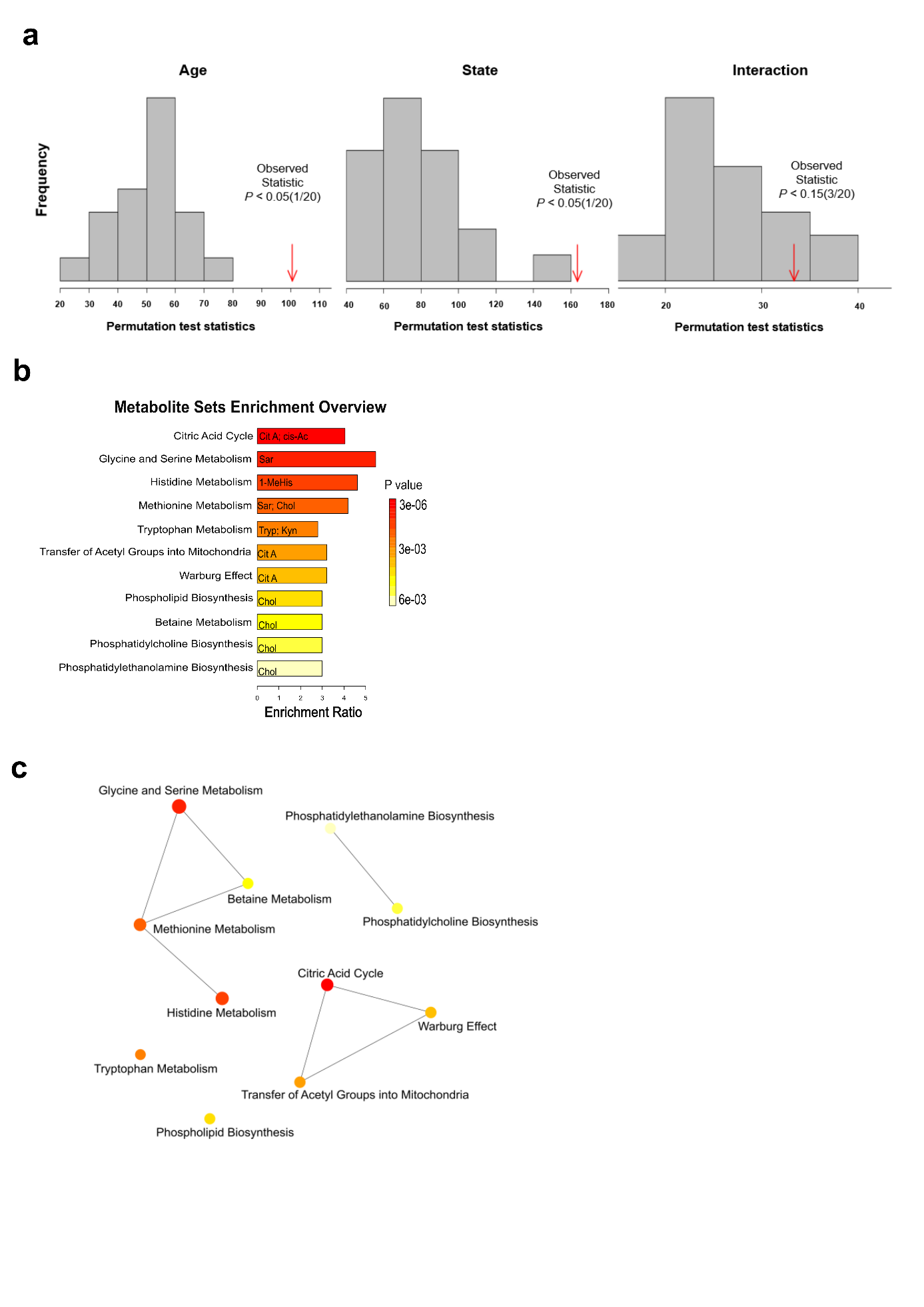
**

**Supplementary Figure 5**

**
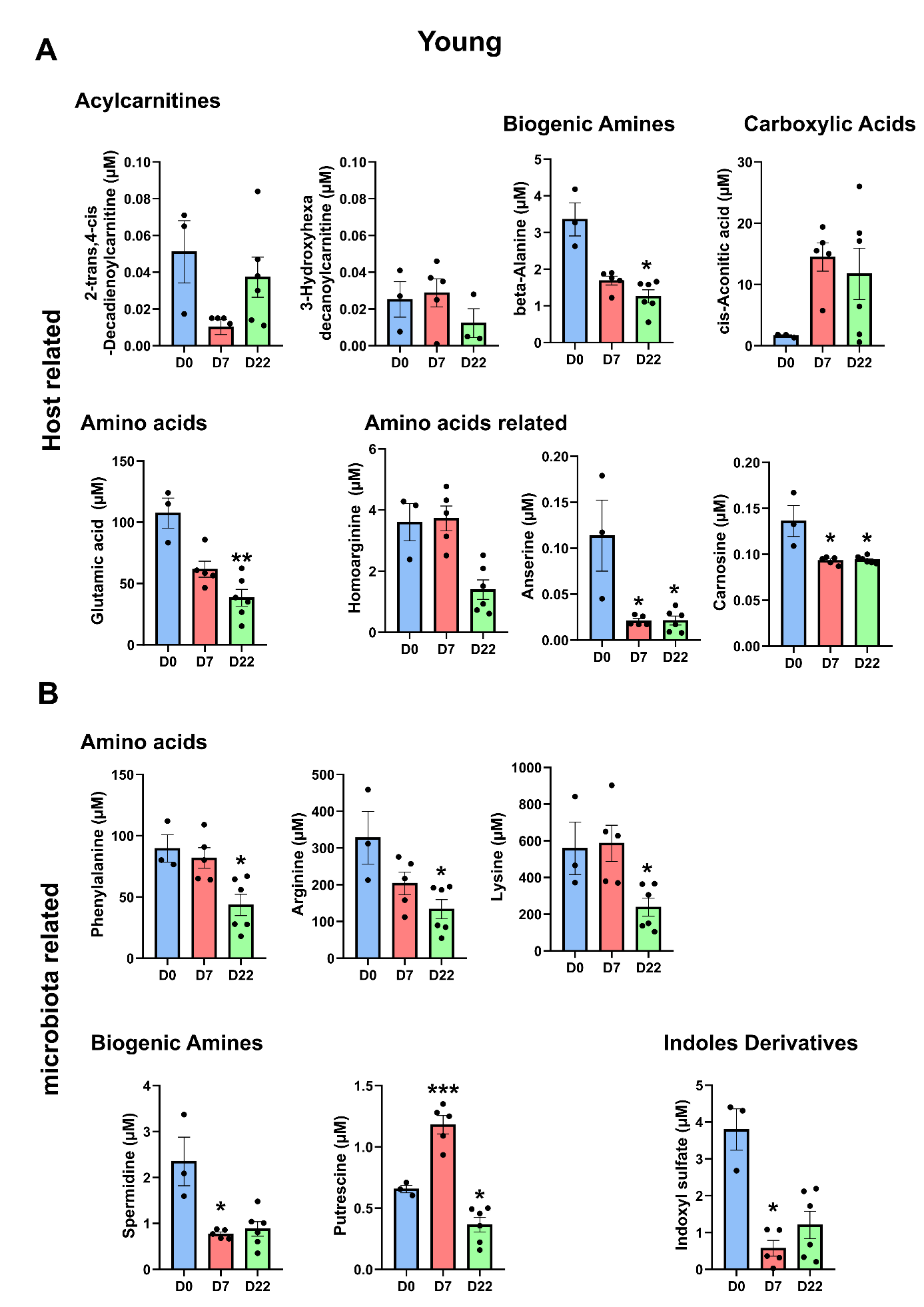
**

**Supplementary Figure 6**

**
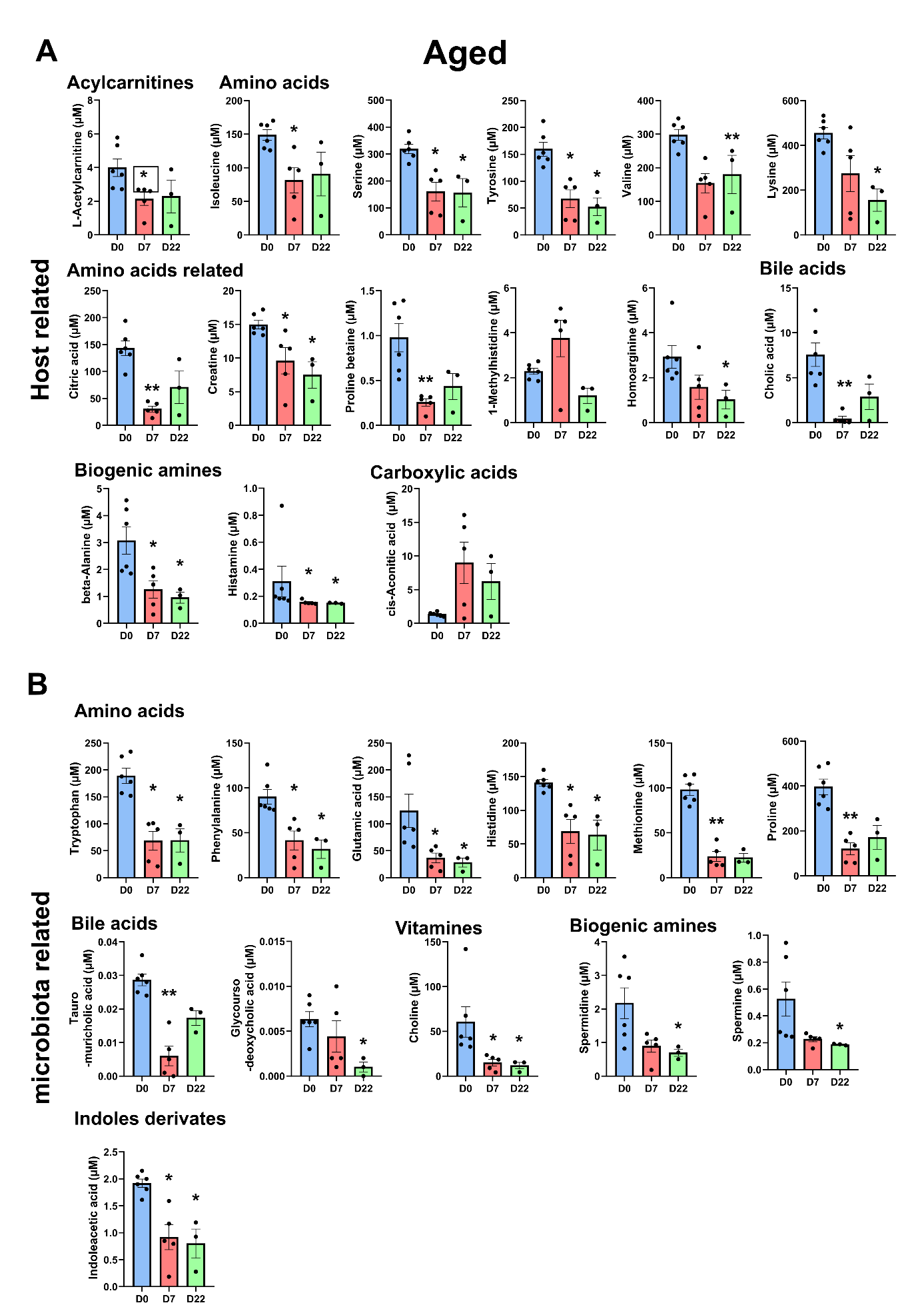
**
